# Evaluating the impact of a multimedia training versus lecture training on attitudes and practices in paediatric nurses in children pain management: A randomized controlled trial

**DOI:** 10.1002/nop2.476

**Published:** 2020-03-18

**Authors:** Afsaneh Arzani, Sousan Valizadeh, Samereh Poorkaremi, Zahra Taheri Ezbarami, Morteza Ghojazadeh

**Affiliations:** ^1^ Non-Communicable Pediatric Disease Research Center Health Research Institute School of Nursing and Midwifery Babol University of Medical Sciences Babol Iran; ^2^ Pediatric Nursing Department Iranian Center for Evidence‐Based Practice School of Nursing and Midwifery Tabriz University of Medical Sciences Tabriz Iran; ^3^ Pediatric Nursing Department School of Nursing and Midwifery Tabriz University of Medical Sciences Tabriz Iran; ^4^ 17 Shahrivar Children's University Hospital Guilan University of Medical Sciences Rasht Iran; ^5^ School of Nursing and Midwifery Guilan University of Medical Sciences Rasht Iran; ^6^ Research Center of Evidence Based Medicine School of Medicine Tabriz University of Medical Sciences Tabriz Iran

**Keywords:** attitudes, children's nurses, evidence‐based practice, pain assessment, pain measurement

## Abstract

**Aim:**

The aim of this study was to evaluate the effect of a multimedia training on pain management attitudes and practices of paediatric nurses.

**Design:**

A single‐blind randomized trial.

**Methods:**

Paediatric nurses (*N* = 120) from a public hospital randomly allocated into two groups, lecturing and multimedia training groups. Nurses in the control group received four one‐hour training sessions. In the intervention group, the same educational content was used as a film with text, audio and animation and presented as a multimedia CD. In both groups, using a questionnaire, we measured pain management attitudes and skills at baseline and then 1 week and 1 month after the training over. Data were analysed in SPSS20 software, and *p* < .05 was considered statistically significant.

**Results:**

The mean of attitude scores of the lecturing group was 104.74 one week after the study and 109.40 one month later versus 74.72. The above‐mentioned scores in the multimedia group were 112.72 and 115.04, respectively, versus 78.70 (*p* < .001). Also, the mean scores of nurses' performance in the lecturing group before, 1 week and 1 month after the study were 12.48, 26.60 and 28.22, respectively, versus 12.58, 28.68 and 28.98, in multimedia group; this difference was statistically significant (*p* < .05).

**Key Practice Points**

The key message of our study is that multimedia training was more effective in improving the attitude and practices of nurses about pain management than lecturing method. By changing their practice and attitude, they will be better and more effective in controlling the pain of children. Otherwise, given the relative advantages and shortage of nursing staff, which could make it difficult for them to attend lectures, it is suggested to use multimedia training method along with other teaching methods, so that more nurses can attend continuing educational courses.

## 1 INTRODUCTION

Pain is the most common side effect of trauma, illness or surgery (Dongara, Nimbalkar, Phatak, Patel, & Nimbalkar, 2017), and 81% of hospitalized children report moderate to severe pain (Van Hulle Vincent, 2007). Lack of pain reduction leads to anxiety, decreased interactions with others, sleep disturbances, movement defects, loss of appetite and restlessness, decreased quality of life and increased health care and hospitalization costs (Jacobsen, Moldrup, Christrup, & Sjogren, 2009). Despite these serious consequences, pain control is not performed at an optimal level (Breivik et al., 2009).

The need to manage hospitalized children's pain effectively is important part of paediatric health care (Clark, 2011) which is initiated by examining and evaluating pain. After diagnosing the type of pain and its effective factors, the treatment plan is provided and medication and non‐pharmacological interventions are done appropriate to the child's condition, and then, the effectiveness of the methods is evaluated (Mondol, Muhammad, & Chowdhury, 2018).

In different studies, the lack of awareness or inadequate practice and the negative attitude of nurses and even physicians about pain measurement and its relief at different ages have been reported as the most important barriers to pain investigation and relief (Gimbler‐Berglund, Ljusegren, & Enskar, 2008; Namnabati, Abazari, & Talakoub, 2012; Salameh, 2018) that can significantly lead to the incorrect pain assessment (Mondol et al., 2018). The results of a study showed that some nurses were ignorant about injecting narcotic drugs because of their negative attitude including fear of addiction, drug tolerance and weakness of the respiratory system and 1/4 of them waited until the patient reported severe pain; then, they provided pharmacotherapy (Noghabi, Soudagar, & Nazari, 2012). The results of a research on nurses in pain management after surgery showed that 70.1% of them had low knowledge, 98.7% had low attitude and 5.2% had very low practice (Ghorbani Moghaddam, Jahanpour, & Hajivandi, 2015). Given the studies' results suggesting the low level of knowledge of nurses about pain management and negative attitude towards the use of analgesics, researchers believe that nurses require periodic and in‐service training concerning the pain of children for its evaluation, assessment and management (Deldar, Froutan, & Ebadi, 2018; O'Neal & Olds, 2016).

Providing effective children's pain management training is essential for nurses. Lecturing is a common, fast, simple and inexpensive teaching method of materials' presentation (Ramlogan, Raman, & Sweet, 2014). However, in the lecturing method, 80% of the training provided is forgotten within 8 weeks (Miller, McNear, & Metz, 2013). So, nowadays, it is recommended to accompany the lecture with other methods and appropriate audiovisual equipment which could improve the efficiency of this method (Salimi, Shahbazi, Mojahed, Ahmadieh, & Dehghanpour, 2007). In this regard, e‐learning in the form of a multimedia approach, whether in the video or CD forms, can be used to convey basic concepts of training in a shorter time (Miller & Metz, 2014). Also, using this method of training can respond to the emerging technology, demand for more education, individuals' tendency for self‐motivation and self‐education, improvement of the quality of education, retention of information, continuity in information acquisition, lack of anxiety during education, full‐time access to educational resources, removing educational spaces and possibility of full‐time access to alternative educational resources and learner‐based approach rather than teacher‐based learning (Masic, 2008).

Essentially, as the need for continuing education and time limitation increases, using electronic methods of training has become more popular (Mahdiyoun, Imanipour, Mojtahedzadeh, & Hosseini, 2015; Qalehsari, Khaghanizadeh, & Ebadi, 2017). Issa et al. (2013) indicated that computer‐based education enhances participants' learning. Also, the meta‐analysis of 27 studies (from 1990–2009) on the effectiveness of computer‐based education in nursing showed the positive effect of this method on knowledge, attitude and practice of nurses with the effect size of 0.43, 0.35 and 0.34, respectively (Roh & Park, 2010). It has been demonstrated that learning in e‐learning is 25% higher than that in traditional classrooms (Tadrisi et al., 2011). One study showed that education through the educational software packages increases the level of knowledge and learning (Nikolaraizi, Vekiri, & Easterbrooks, 2013). It is important that nursing has adequate training in paediatric health. In Iran, few studies have been conducted on the results of using multimedia training and the effectiveness of modern approaches rather than traditional methods in paediatric pain management education. The purpose of our study was to examine the effectiveness of multimedia versus lecture training on the attitudes and practices of nurses in paediatric pain management.

## 2 METHODS

### 2.1 Design and aim

This single‐blind, randomized, pre‐ and post‐test equivalent control group design was conducted to examine the effectiveness of a multimedia versus lecture training on the attitudes and practices of paediatric nurses. The registration code for this study on the clinical trial website is IRCT201607314613N21.

### 2.2 Sample size and participants

A pilot study was done to calculate the sample size, and the mean (standard deviation, *SD*) and score of the participants' practice were obtained in two groups as 11.24 (*SD*, 3.6) and 13.62 (*SD*, 4.1), respectively. Considering the type I error value of *α* = .05 and with 80% power, the sample size of 43 was obtained per group. To increase the credibility of the samples, 20% was added to the sample size. Finally, 60 samples were determined for each group.

Nurses were selected from a paediatric teaching hospital, affiliated to Gilan University of Medical Sciences located in the capital city of Rasht, Iran. The hospital serves a large mixed urban/rural region and takes referrals for paediatric specialist care in Gilan province. The inclusion criteria for nurses included a minimum 6‐month work experience in the paediatric ward, knowing how to use computer and multimedia CDs and having access to computers both at home and work. The exclusion criteria included unwillingness to continue participation and not completing questionnaires in the due time. One hundred and twenty nurses were participated in first phase of this study. However, finally, 50 nurses were analysed per group, participated in second phase of this study, resulting in a attrition rate of 17% with withdrawals from the education programme (due to the lack of participation of 10 nurses in the lecture group and in the multimedia CD training group, lack of completion of the questionnaires by four nurses and lack of software study by six nurses).

### 2.3 Questionnaire

To assess the nurses' attitude, the questionnaire prepared by Alavi et al. (2008) was used. It included 20 items with 6‐point Likert scale (from “Totally agree” with the score of 6 to “Totally disagree” with the score of 1), and reverse scoring was used for negative items. Therefore, the attitude score varied from at least 20 to the maximum of 120; if the score increased, the nurses' attitude would be more positive.

The nurses' practice was assessed using Alavi et al.'s (2008) checklist of nurses' performance in pain management and included questions about the evaluation and management of pain (32 items) and two case studies (each with two questions and the total of four scores) with the minimum score of 0 and maximum of 36. Accordingly, if the score increased, the nurses' practice would be more optimal.

These questionnaires were previously used in the studies conducted by Noghabi et al. (2012) and Alavi et al. (2008) in Iran after verifying the validity and reliability of the tools. To further ensure the credibility, for assessing the face and content validity of the tools in this study, the questionnaire was submitted to 10 faculty members of Tabriz University of Medical Sciences, including paediatric nursing professors and paediatricians, and their corrective suggestions were applied.

To assess the reliability of the questionnaires, a preliminary study was conducted on 20 nurses working in a paediatric hospital in Tabriz. Cronbach's alpha was used to calculate the internal reliability of the instrument. Results for the attitude questionnaire were 0.81 and 0.74, respectively. These individuals were not included in the final sample of the study.

### 2.4 Description of training content

To design and produce multimedia CDs, first, a review of the related content was performed and the educational content was developed; then, by using Mayer's 10 principles of multimedia design, evaluation criteria for educational materials and the checklist for evaluating educational materials were modified. This programme was composed of text, audio, image, film and animation, and the Flash software was employed for its development.

### 2.5 Data collecting

After obtaining an ethical licence from Regional Ethics Committee of Tabriz University of Medical Sciences, with no. IR. TBZMED.REC.1395.611, registering the study on IRCT website with no. IRCT201607314613N21 and obtaining permission to conduct the study from the deputy of Gilan Universities of Medical Sciences to access the research units, the researcher visited the above centre to collect the data required after coordination with the hospital authorities.

Data collection was undertaken from December 2017–February 2018. First, 120 nurses were selected based on the inclusion criteria and the purpose of the study was explained to them. If the nurses intended to participate in the study, the informed consent was obtained and nurses completed demographic characteristics, attitude and practice questionnaire about pain management in children. Then, based on the attitude and practice scores, the samples were randomly assigned to two groups of 60 (lecturing or multimedia training groups) using Rand List 1.2 software.

In the lecture group, with previous invitations, nurses received four one‐hour training sessions at the amphitheatre of the health centre by one of the researchers using PowerPoint and whiteboard.

In the multimedia group, nurses received a multimedia CD that the same educational content was used as a film with text, audio and animation. These CDs were capable of running on a PC and laptop. The training process was as distant and individual education at home or at their workplace. The first and second post‐tests were administered 1 week and 1 month later after finish intervention sessions, respectively, in both groups using the same attitude and practice questionnaires.

### 2.6 Data analysis

Data were analysed by SPSS version 21, and *p* < .05 was considered statistically significant. Socio‐demographic characteristics were reported as numbers (%) for qualitative and mean (*SD*) for quantitative variables, and the comparability of groups was examined using the chi‐square test and independent sample *t* test. To compare the effect of lecture and multimedia on the attitude and practice of nurses, repeated measures analysis of variances was used by modifying the basic scores and paired sample *t* test, appropriate. Nurses were not blind to the randomization process. Data analyzer was blinded to the data files and study process.

### 2.7 Ethical aspects

Our research protocol was approved by the Ethics Committee of Tabriz University of Medical Sciences (Number IR.TBZMED.REC.1395.611). We also received authorization from the Gilan University of Medical Sciences to conduct the study. The informed oral consent of the nurses was sought prior to completing the questionnaires. Data were collected anonymously at two time periods and were only used for the study.

## 3 RESULTS

Our study sample included 100 nurses amongst whom 74% (37), 60% (30) were married, 94% (47/47) were bachelor degree, 6% (3/2) master degree in nursing, respectively, in multimedia and lecture groups. The average age for nurses was 36.1 (*SD*, 5.1) and 36.3 (*SD*, 1.8) years, most of the nurses were working on rotational shifts 64% (32), 68% (32), they were official employees and did not undergo pain training, respectively, in multimedia and lecture groups. There was no difference between the socio‐demographic characteristics of nurses participating in these two groups.

Table 1 shows that the mean scores of attitude in both lecture and multimedia groups were significantly different, so that the mean score of the attitude of the lecture group in the pre‐test was 74.72, while the scores in the first and second post‐tests, that is 1 week and 1 month later, were 104.70 and 109.40, respectively. In the multimedia group, the mean score of attitude in the pre‐test was 78.70, while the scores in the first and second post‐tests, that is 1 week and 1 month later, were 112.72 and 115/04, respectively. The mean difference in the lecture group was 34.02 between pre‐test and the first post‐test and 36.34 between the first and second post‐tests. The mean difference in the multimedia group in the pre‐test and the first stage of post‐test and in the pre‐test and the second stage of post‐test was 29.98 and 34.68, respectively. The results of repeated measures ANOVA to compare the mean scores of nurses' attitudes in both lecture and multimedia groups showed that the time effect was not statistically significant on the attitude of the participants in the study, but the interaction of time group was statistically significant.

**Table 1:** Comparison of nurses' attitude scores about pain management in children in two groups at different times during the study (*N* = 30 in each group)

Group	Attitude scores		
	At the beginning of the study	1 week after intervention	Reminder 1 month after the intervention
Lecture group (*N* = 50) M ± *SD*	74.72 ± 17.09	104.72 ± 10.11	109.40 ± 6.88
Multimedia group (*N* = 50) M ± *SD*	78.70 ± 15.68	112.72 ± 6.39	115.04 ± 4.81
Statistical test result			
Group factor	*F* = 33.137	*p* = .000	*df* = 1
Time factor	*F* = −0.78	*p* = .781	*df* = 1
Interaction of group and time	*F* = 1,528.24	*p* = .000	*df* = 1

*F* = repeated measure Analysis of Variances (ANOVA).

Abbreviations: M, mean; *SD*, standard deviation.

Table 2 shows that the mean scores of the nurses' practice of the lecture group in the pre‐test were 12.48, while the scores in the first and second post‐tests, that is 1 week and 1 month later, were 26.60 and 28.22, respectively. In the multimedia group, the mean scores of the nurses' practice were 12.58, 28.68 and 28.98, respectively. The mean difference in the lecture group in the pre‐test and the first stage of post‐test and in the pre‐test and the second stage of post‐test was 14.12 and 15.74, respectively. In the lecture group in the pre‐test and the first stage of post‐test and in the pre‐test and the second stage of post‐test, it was 16.10 and 16.40, respectively. The results of repeated measures ANOVA for comparing the mean scores of nurses' practices both in lecture and multimedia groups showed that time and group factors were statistically significant for the practices of the participants (*p* = .006), but there was no statistically significant difference in the interaction of time group (*p* = .053). On the other hand, the practice of nurses in two groups of lecture and multimedia had a statistically significant difference (*p* = .026).

**Table 2:** Comparison of nurses' practices scores about pain management in children in two groups at different times during the study (*N* = 30 in each group)

Group	Practice scores		
	At the beginning of the study	1 week after intervention	Reminder 1 month after the intervention
Lecture group (*N* = 50) *M* ± *SD*	12.48 ± 6.35	26.60 ± 5.19	28.22 ± 3.23
Lecture group (*N* = 50) *M* ± *SD*	12.58 ± 5.50	28.68 ± 2.73	28.98 ± 2.36
Statistical test result			
Group factor	*F* = 5.115	*p* = .026	*df* = 1
Time factor	*F* = 7.933	*p* = .006	*df* = 1
Interaction of group and time	*F* = 3.840	*p* = .053	*df* = 1

*F* = repeated measure Analysis of Variances (ANOVA).

Abbreviations: M, mean; *SD*, standard deviation.

## 4 DISCUSSION

Findings of our study showed that although nurses' attitude and practice level increased in both educational methods, this level was higher in the multimedia method and the mean difference between these two groups was statistically significant. In the recall stage (1 month after the intervention), nurses' learning in the multimedia method was higher than the lecture method. In line with our results, Mollazadeh, Kameli, Jafari chogan, Mirhosseini, and SHoja (2014) found that multimedia training method had longer persistence in nursing students than the lecture method in learning principles and techniques. Also, the results of other studies showed that although the level of learning of nurses in triage training was improved in both training methods, the recall in the multimedia group was more than that in the lecture group (Tadrisi et al., 2011).

The results of this study and other researchers have demonstrated that multimedia training has a more statistically significant and lasting persistence effect than single interventional approaches in behaviour, belief and attitude change, as the learner uses various senses to learn in integrated methods and have more opportunities to think deeply on the content and exchange ideas with colleagues (Alavi et al., 2008; Mollazadeh et al., 2014; Perz, Thompson, Schaefer, & Patel, 2010). Also, information with image, text and audio, 24‐hr access to learning content in different places, engaging the learner in the learning activity and increasing motivation can have a statistically significant effect on meaningful learning (Hsu, 2012). Due to the hierarchical layout of concepts in human memory, retrieval of the learned information in a multimedia approach is performed better; so, the material will remain in memory for a long time.

Consistent with our results, Varaei, Mamashli, Ghiyasvandian, and Bahrani (2016) and Bahreini, Bijani, Rahmati, and Shahamat (2011) have also obtained similar results, so that nurses' practice in the field of safe injection and exposure to sharp and infected objects is enhanced using the multimedia training method. But results of Valizadeh, Feizalahzadeh, Avari, and Virani (2016) indicated that the use of educational software has no statistically significant effect on nursing students' drug knowledge or medicinal calculation ability. However, e‐learning programmes can reduce lecture time and cost of repeated topics (e.g. medication), suggesting that it can be an effective component in nurse education programmes.

Traditional methods such as lectures do not have the necessary effectiveness in improving professional practice (Qalehsari et al., 2017). Nursing training can maintain its dynamism, which breaks the boundaries of time and space and proceeds into the development and innovation of modern methods of teaching and learning (Tajari & Tajari, 2011).

Concerning the attitude of nurses about the management of pain in children, our study also showed that attitude change was statistically significant in both groups using the multimedia training method. However, the findings of Kardan et al. did not show any statistically significant difference in the attitude change in the cardiac nurses in the care for patient with a pacemaker care using both methods of lecture and multimedia training, which meant that multimedia training could be as much effective as the lecture method. Therefore, using multimedia training method can be provided as an appropriate strategy to meet the increasing demand for training and the nurses' fatigue due to numerous work shifts and time and space constraints (Kardan Barzoki, Bakhshandeh, Nikpajouh, Elahi, & Haghjoo, 2016).

The effectiveness of the multimedia training method in the practice and attitude of nurses in our study was estimated to be 0.1 and 0.04, respectively. But the Roh meta‐analysis about the effectiveness of computer‐based nursing training had a positive effect on the knowledge, attitude and practice of nurses with the effects of 0.43, 0.35 and 0.34, respectively (Roh & Park, 2010).

Regarding the point that today's multimedia training method in continuous and lifelong education is considered important and necessary in all occupations and professions, it is recommended that special attention be paid to this training method in the nursing profession. In this regard, research has shown that multimedia training method provides more convenient facilities for learners in terms of time, place and financial issues. However, Kusi Amponsah et al. (2020) Study’s shows that the sampled nurses and nurse managers indicated diverse preferences on the nature of the paediatric pain educational programme based on their personal choices and working dynamics. So that the education provided would be congruent With nurse preferences.

### 4.1 Limitations

The limitations of our study included inaccurate responses of nurses to questions and uncertainty about the use of educational CDs by nurses in the multimedia group, which could affect the results.

## 5 CONCLUSION

Our study indicated that both lecture and multimedia training methods promote the level of nurses' attitude and practice in pain management. However, in the multimedia method, both level of data and retrieval of data were higher. Given the relative advantages and shortage of nursing staff, which could make it difficult for them to attend lectures, it is suggested to use multimedia training method along with other teaching methods, so that more nurses can attend continuing educational courses.

## CONFLICT OF INTEREST

The authors declare that there is no conflict of interest.

## AUTHOR CONTRIBUTIONS

SV, AA: Study design. SP, SV: Data collection. SP, MG: Data analysis. SP, SV, AA, ZTE, MG: Drafting of the manuscript.

## ETHICAL APPROVAL

The study was approved by code of ethics with number: IR.TBZMED.REC.1395.611.
